# Novel technique using pancreatic duct stent facilitates difficult biliary cannulation in patients with Roux‐en‐Y anatomy (with video)

**DOI:** 10.1002/jgh3.12227

**Published:** 2019-07-19

**Authors:** Yuki Tanisaka, Shomei Ryozawa, Masafumi Mizuide, Akashi Fujita, Maiko Harada, Tomoya Ogawa

**Affiliations:** ^1^ Department of Gastroenterology Saitama Medical University International Medical Center Hidaka Japan

**Keywords:** endoscopic retrograde cholangiopancreatography, pancreatic duct stent, surgically altered gastrointestinal anatomy

## Abstract

Endoscopic retrograde cholangiopancreatography (ERCP) in patients with surgically altered gastrointestinal anatomy has been reported to be useful. However, selective biliary cannulation through the papilla is difficult in cases with surgically altered gastrointestinal anatomy. Herein, we report a successful biliary cannulation using a pancreatic duct (PD) stent in patients with Roux‐en‐Y anatomy. A 70‐year‐old man who underwent total gastrectomy with Roux‐en‐Y anatomy was admitted to our hospital with jaundice due to recurrence of gastric cancer. ERCP was performed for biliary drainage. We approached the papilla using a short‐type single‐balloon enteroscope (SIF‐H290; Olympus Medical Systems). Because the papilla was positioned tangentially, it was difficult to adjust the catheter in the direction of the bile duct. As only a PD could be cannulated, we placed a guidewire in the PD. Although we attempted the double‐guidewire technique using a guidewire placed in PD, selective biliary cannulation was difficult. Therefore, we placed a PD stent 5Fr‐5cm (Geenen, Pancreatic Stent Sets, Cook Medical, Bloomington, IN, USA) to assist biliary cannulation. We inserted a catheter crossing the PD stent. With this, selective biliary cannulation was successful. We successfully performed selective biliary cannulation using the PD stent as we were able to fix the papilla, straighten the common channel and the axis of the bile duct, and not restrict scope movement by not using the PD guidewire placement method. This novel technique using a PD stent appears to be useful in patients with surgically altered gastrointestinal anatomy.

## Graphical abstract

This study presents the case of a 70‐year‐old man who underwent total gastrectomy with Roux‐en‐Y anatomy. It was difficult to adjust the catheter in the direction of the bile duct despite using double‐guidewire technique. We placed a 5Fr‐5cm pancreatic duct (PD) stent to assist biliary cannulation. We inserted a catheter crossing the PD stent. By doing so, selective biliary cannulation was successful.

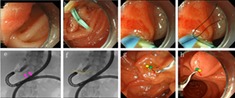

## Introduction

When performing endoscopic retrograde cholangiopancreatography (ERCP) in patients with surgically altered gastrointestinal anatomy, postoperative adhesion and special anatomical characteristics, such as distance or strong curvature of the intestine, increase the difficulty of reaching the papilla or choledochojejunal anastomosis using a conventional endoscope. Similarly, using a duodenoscope and performing bile duct cannulation and subsequent treatments are technically more difficult in patients with altered anatomy than in those with normal anatomy. A short‐type single‐balloon enteroscope, with a working length of 152 cm and working channel of 3.2‐mm, was developed to increase the number of devices that can be used during ERCP in patients with surgically altered gastrointestinal anatomy and was reported to be useful.^1^, ^2^, ^3^, ^4^, ^5^ However, selective biliary cannulation through the papilla is difficult in patients with surgically altered gastrointestinal anatomy than in those with normal anatomy because the appearance of the papilla is a reversal of its usual appearance in normal anatomy, the position is frequently tangential, and a forward‐viewing endoscope without an elevator is required. Recently, the use of a pancreatic duct (PD) stent to facilitate selective biliary cannulation in patients with normal anatomy was described.^6^, ^7^, ^8^ Here, we report a case of successful biliary cannulation using a PD stent in patients with Roux‐en‐Y anatomy.

## Case Report

**Figure 1:**
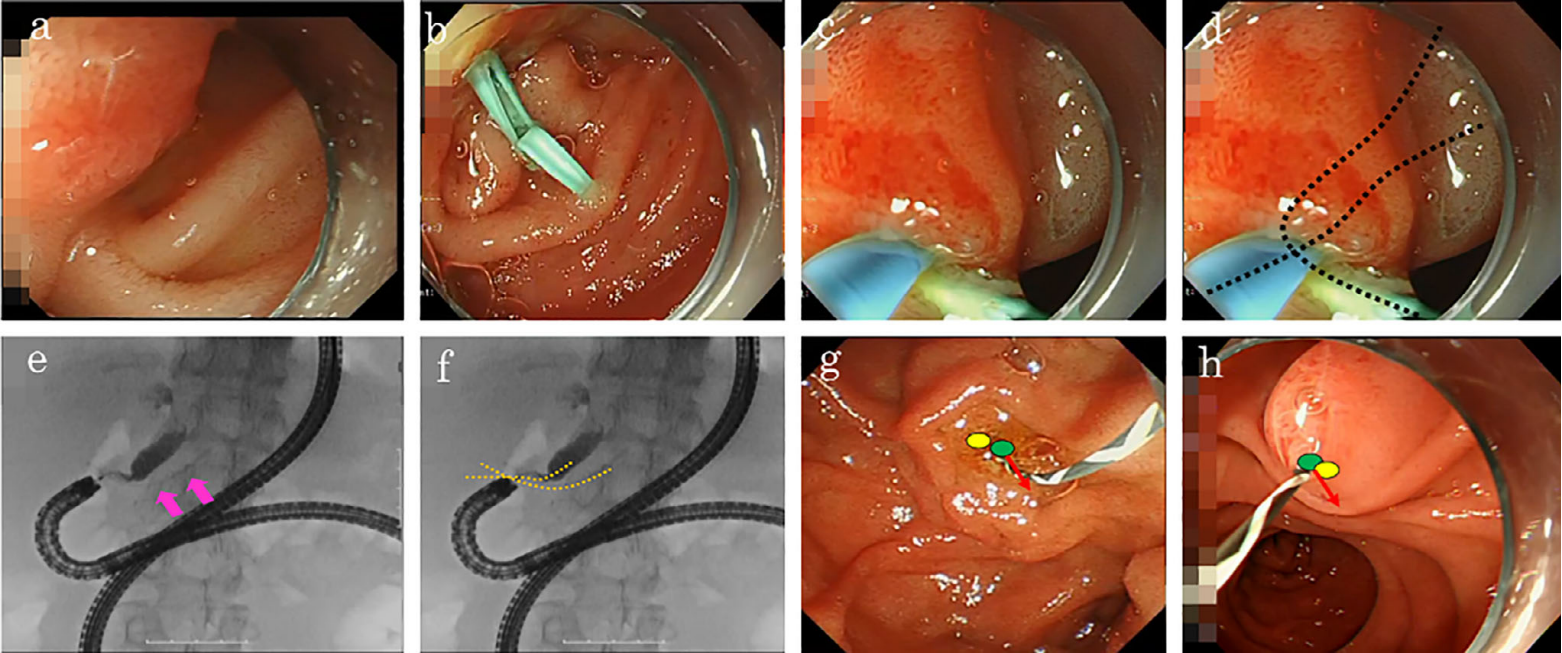
(a, b) Endoscopic findings. As the papilla was positioned tangentially, it was difficult to adjust the catheter in the direction of the bile duct despite using the double‐guidewire technique. We placed a pancreatic duct (PD) stent to assist biliary cannulation. (c, d) The catheter was inserted crossing the PD stent. (e, f) Endoscopic retrograde cholangiopancreatography findings. Fluoroscopy demonstrated selective biliary cannulation using the PD stent (pink arrow). The catheter was inserted crossing the PD stent. (g, h) PD guidewire placement method (green circle: PD, yellow circle: bile duct). (g) In patients with normal anatomy: The guidewire placed in the PD acts in a direction away from the bile duct by gravity or scope (red arrow) so that the bile duct and the PD are separated. (h) In patients with surgically altered gastrointestinal anatomy: As the guidewire placed in the PD acts in the same direction as the bile duct (red arrow), the bile duct and PD cannot be properly separated.

A 70‐year‐old man who underwent total gastrectomy with Roux‐en‐Y anatomy was admitted to our hospital with jaundice due to the recurrence of gastric cancer. The stricture was located at the hilar bile duct. ERCP was performed for biliary drainage (Video Clip S1). We approached the papilla using a short‐type single‐balloon enteroscope (SIF‐H290; Olympus Medical Systems, Tokyo, Japan). After reaching the papilla, a standard ERCP cannula (ERCP catheter, MTW Endoskopie, Wesel, Germany) and a 0.025‐inch angle‐tip guidewire (Visiglide2, Olympus Medical Systems) were used for selective biliary cannulation. However, because the papilla was positioned tangentially, it was difficult to adjust the catheter in the direction of the bile duct despite using a distal attachment cap (D‐201‐10704; Olympus Medical Systems) and a tool that can be rotated (Swingtip, Olympus Medical Systems) (Fig. 1a). As only a PD can be cannulated, we placed a guidewire in the PD. Although we attempted the double‐guidewire technique using a guidewire placed in the PD, selective biliary cannulation was difficult. Therefore, we placed a 5‐Fr and 5‐cm PD stent (Geenen, Pancreatic Stent Sets, Cook Medical, Bloomington, IN, USA) to assist biliary cannulation (Fig. 1b). We inserted a catheter crossing the PD stent (Fig. 1c,d). With this, selective biliary cannulation was successful (Fig. 1e,f). After selective biliary cannulation, we removed the PD stent and finally placed a metallic stent above the papilla for biliary drainage.

## Discussion

In cases of difficult biliary cannulation, the PD guidewire placement (double‐guidewire technique) and PD stent placement methods are useful to fix a papilla and straighten the common channel and the axis of the bile duct. We believe that the PD stent placement method is useful in patients with surgically altered gastrointestinal anatomy because, in patients with normal anatomy, the guidewire placed in the PD acts in a direction away from the bile duct due to gravity or scope, such that the bile duct and PD are separated. On the other hand, in patients with surgically altered gastrointestinal anatomy, as the guidewire placed in the PD acts the same direction as the bile duct, the bile duct and PD cannot be properly separated (Fig. 1g,h). Furthermore, unlike the double‐guidewire technique, the PD stent placement method did not restrict scope movement, thus facilitating the approach to the papilla. Although we removed the PD stent in the present case, and post‐ERCP acute pancreatitis did not occur, it is preferable to not remove a PD stent if the PD is damaged due to procedures.

We successfully performed selective biliary cannulation using the PD stent as we were able to fix the papilla, straighten the common channel and the axis of the bile duct, and not restrict scope movement by not using the PD guidewire placement method. This novel technique using a PD stent appears to be useful in patients with surgically altered gastrointestinal anatomy.

## Supporting information


**Video Clip S1** As the papilla was positioned tangentially, it was difficult to adjust the catheter in the direction of the bile duct despite using the double‐guidewire technique. We placed a pancreatic duct (PD) stent to assist biliary cannulation. The catheter was inserted crossing the PD stent. Fluoroscopy demonstrated selective biliary cannulation using the PD stent.
